# Autophagy and Its Interaction With Intracellular Bacterial Pathogens

**DOI:** 10.3389/fimmu.2018.00935

**Published:** 2018-05-23

**Authors:** Mariana da Silva Siqueira, Renato de Moraes Ribeiro, Leonardo H. Travassos

**Affiliations:** Laboratory of Immunoreceptors and Signaling, Immunobiology Program, Institute of Biophysics Carlos Chagas Filho, Federal University of Rio de Janeiro, Rio de Janeiro, Brazil

**Keywords:** autophagy, infection, *Mycobacterium tuberculosis*, *Shigella flexneri*, *Listeria monocytogenes*, *Salmonella typhimurium*, *Streptococcus pyogenes*, *Legionella pneumophila*

## Abstract

Cellular responses to stress can be defined by the overwhelming number of changes that cells go through upon contact with and stressful conditions such as infection and modifications in nutritional status. One of the main cellular responses to stress is autophagy. Much progress has been made in the understanding of the mechanisms involved in the induction of autophagy during infection by intracellular bacteria. This review aims to discuss recent findings on the role of autophagy as a cellular response to intracellular bacterial pathogens such as, *Streptococcus pyogenes, Mycobacterium tuberculosis, Shigella flexneri, Salmonella typhimurium, Listeria monocytogenes, and Legionella pneumophila*, how the autophagic machinery senses these bacteria directly or indirectly (through the detection of bacteria-induced nutritional stress), and how some of these bacterial pathogens manage to escape from autophagy.

## Introduction

Autophagy is a homeostatic and highly conserved survival mechanism in which portions of the cytoplasm such as long-lived proteins and damaged organelles are sequestered in double-membrane vesicles (called autophagosomes). Then, autophagosomes fuse with lysosomes, leading to the degradation of the sequestered content and recycling of functional blocks for anabolic processes, especially during nutrient shortages (1). Indeed, for many years, autophagy was mainly considered as a breakdown process to degrade macromolecules to generate energy during nutrient deprivation. To date, three types of autophagy have been described, chaperone-mediated autophagy, microautophagy, and macroautophagy (1). Here, we discuss the interaction of the best-characterized type of autophagy (macroautophagy, hereafter autophagy), with intracellular bacterial pathogens, a process designated xenophagy.

The first report demonstrating induction of autophagy by bacteria was published in 1984. In this study, Rikihisa described the presence of vesicles containing glycogen granules and rickettsiae in Guinea pig polymorphonuclear (PMNs) cells infected with the bacteria (2). Despite this initial study, it was only after the studies from Nakagawa et al. and Gutierrez et al. that autophagy was regarded as an important cell autonomous arm of the innate immune system against intracellular bacteria. In their seminal and independent studies, Nakagawa et al. and Gutierrez et al. demonstrated a crucial role for autophagy in the sequestration and degradation of group A *Streptococcus* (GAS) and *Mycobacterium bovis* BCG, respectively (3, 4). Since then, an amazing number of elegant studies have demonstrated a key role of autophagy in the control of infection by different bacterial pathogens and also how some of these most well-succeeded pathogens circumvent or even use autophagy to establish replicative niches inside different cell types (5–7).

### The Autophagosome Formation Core Machinery

Possibly one of the most exciting areas in the field of autophagy, the mechanisms involved in the formation of autophagosomes, the hallmark of this process, have been the focus of many research groups. Morphologically, autophagy begins with the formation of a cup-shaped double-membrane structure that surrounds the cargo. Upon its complete closure, the phagophore is now called an autophagosome, a transient organelle that delivers its content for degradation in lysosomes (8). After extensive work from several groups, the proteins that participate in autophagosome biogenesis can be categorized into complexes that take place in different steps of the autophagosome formation (1). Below, we will summarize the different steps of the autophagic process and the major protein groups that take part in each step of the whole process and discuss critical findings linking these proteins with bacterial-induced autophagy. For extensive literature on autophagosome formation machinery, please refer to Suzuki et al. (9) and Yin et al. (10).

### Signal Induction

#### The ULK Complex and Autophagy Induction

The uncoordinated-51-like kinase (ULK1) complex comprising ULK1, ATG13, FAK family kinase-interacting protein of 200 kDa (FIP200), and ATG101 is responsible for sensing changes in nutrient status within the cell. Its activation is instrumental in the initiation of autophagy. This complex works downstream mammalian target of rapamycin complex 1 (mTORC1) and under, nutrient-rich conditions is phosphorylated by mTOR, which inhibits ULK1 recruitment to the phagophore assembly site (PAS).

Under nutrient starvation, however, mTORC1 is inactivated, and ULK1 is released, allowing FIP200 phosphorylation and translocation of the complex to PAS for the recruitment of ATG proteins, required for autophagosome formation (11). Interestingly, components of the ULK complex have also been shown to target bacterial vacuoles during infection with intracellular bacteria (12). This is the case of FIP200 during infection with *Salmonella typhimurium*. Experiments performed by Kageyama et al. suggest that this protein is recruited to the vicinity of vacuoles containing *S. typhimurium*. See below for more detailed information regarding autophagy induced by this pathogen.

### Nucleation

#### Class III Phosphatidylinositol 3-Kinase (PtdIns3K) Complex and Trafficking of Atg9 for Autophagosome Nucleation

The class III PtdIns3K complex consisting of Beclin 1, ATG14L, phosphoinositide 3-kinase regulatory subunit 4 (PIK3R4) are recruited to PAS to initiate phagophore membrane nucleation through the activation of PtdIns3-kinase class III (PtdIns3KC3). As a result, PtdIns3P is generated at this site, and the PtdIns3P-binding protein WD-repeat domain phosphoinositide-interacting 1 (WIPI1) and 2 (WIPI2) are recruited to the PAS, allowing ATG proteins to be recruited later on (13). Mammalian Atg9 (mAtg9) is another protein required for the assembly of phagophore, although its role is still not completely understood. It has been demonstrated that mAtg9 is not necessary for LC3 recruitment to phagophore, but essential for its generation following infection with *Salmonella typhimurium* (12).

### Expansion

#### Ubiquitin-Like Conjugation Systems and Autophagosome Expansion

Pivotal for the formation of autophagosomes are two ubiquitin-like conjugation systems: Atg8/LC3 and Atg12. The Atg8/LC3 system modifies the core autophagy protein microtubule-associated 1 light chain 3 (LC3). LC3 has a diffuse cytosolic distribution pattern and is cleaved at its C-terminus by the cysteine protease Atg4 to form LC3-I, which has a C-terminal glycine residue. Upon autophagy induction, LC3-I is sequentially modified by the E1-like enzyme Atg7 and the E2-like enzyme Atg3 to form LC3-II after the conjugation of LC3-I to phosphatidylethanolamine (PE). This lipidated form of LC3 is attached to both outer and inner phagophore membrane being eventually removed from the autophagosomal membrane by Atg4 before the fusion with late endosomes/lysosomes (1, 14). In the Atg12 conjugation system, Atg5 and Atg12 proteins form a complex through the covalent binding of Atg12 to the C-terminus of Atg5 in a reaction involving Atg7 and Atg10. Then, the scaffold protein Atg16L1 is conjugated to Atg5 *via* its N-terminus, forming the 800 kDa Atg12–Atg5–Atg16L1 complex. It has been proposed that the Atg16L1 complex works as an E3-like enzyme to target LC3-I to its membrane site of lipid conjugation (15). Data from the literature suggest that these two systems work coordinately as in Atg3-deficient cells, where no LC3-II is found, Atg12–Atg5 conjugation is dramatically reduced (16).

Alternative (non-canonical) forms of autophagy have been identified and reported to target invading bacteria (17–19). In this review, however, we will focus on xenophagy and its implication in intracellular bacterial infections.

### Cargo Selection During Infection With Bacterial Pathogens

Invasion of host cytosol by bacteria imposes a significant challenge to homeostasis and triggers several cellular and immune responses such as proinflammatory cascades and cell-autonomous in an attempt to control of bacterial replication, such as xenophagy.

In addition to the steps discussed above, autophagy has an additional and essential step that is cargo selection. One of the central questions regarding xenophagy relates to its specificity and how autophagy machinery specifically recognizes bacteria. This is of major importance as xenophagy, which eventually aims to reduce not only bacterial load but also prevent cellular stress resulting, for instance, from bacteria-induced amino acid starvation (see later in this review). To explain the central mechanisms involved in the selection of intracellular bacteria by the autophagy machinery, we will focus on bacterial models that helped us shape the field.

#### Mycobacterium tuberculosis

*Mycobacterium tuberculosis* is the causative agent of tuberculosis (TB), possibly one of the oldest human pathogens and still among the top 10 causes of death worldwide (20). *M. tuberculosis* is a non-motile and facultative intracellular pathogen of macrophages. In this regard, the infection of alveolar macrophages is a crucial requisite toward the establishment of a successful replicative niche. Experiments using mice depleted for resident alveolar macrophages have shown that these animals become protected from *M. tuberculosis* (21). One of the main features of TB pathogenesis is the ability of *M. tuberculosis* to survive within alveolar macrophages through the interference with phagolysosome biogenesis (3, 22).

**Figure 1:**
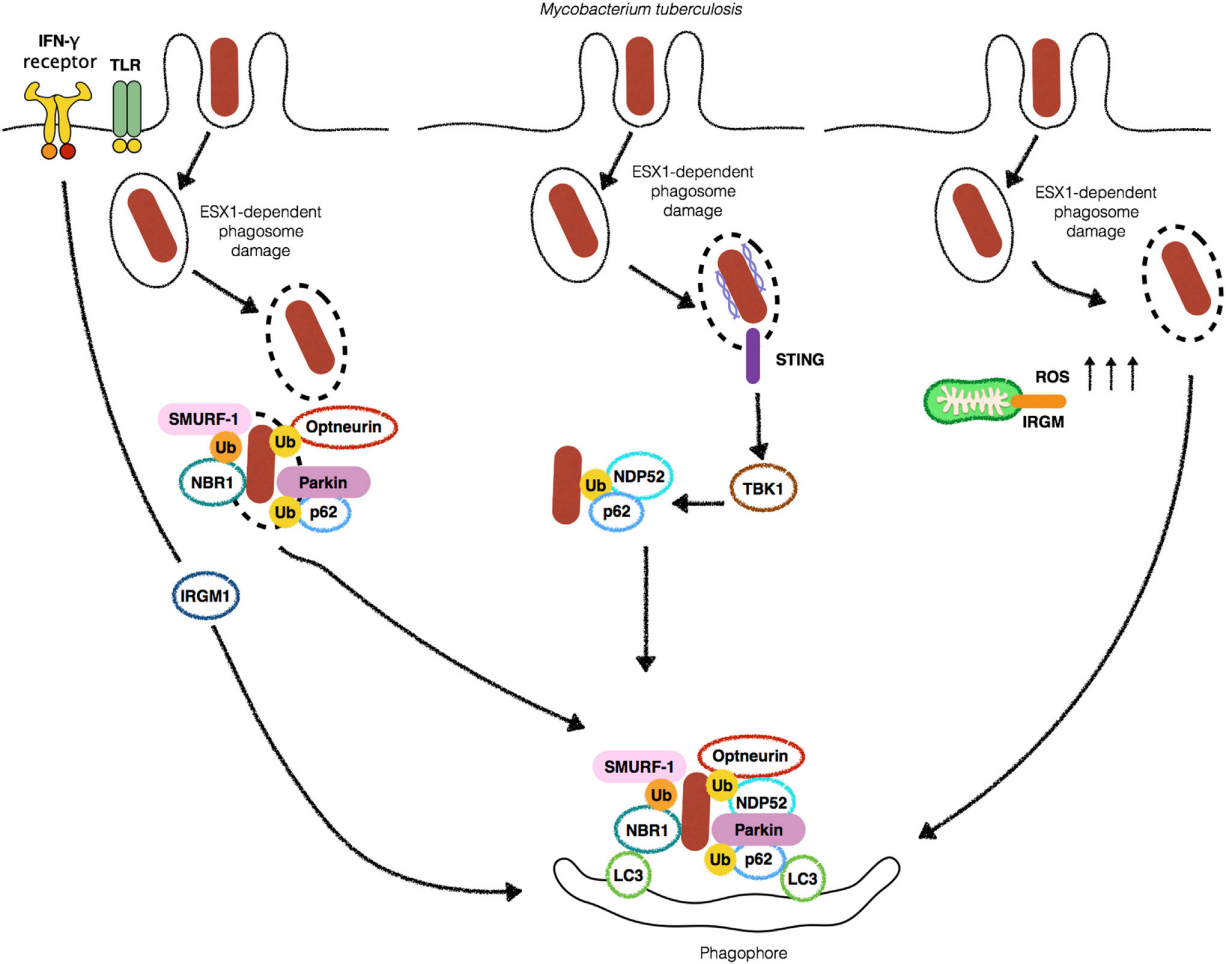
Autophagy targets *Mycobacterium tuberculosis* (Mtb) by different mechanisms. Stimulation with IFN-γ or Toll-like receptors (TLRs) ligands leads to an increase in the localization of (Mtb) into autophagosomes (left). 6-kDa early secretory antigenic target (ESAT-6) secretion system 1 (ESX-1)-induced phagosomal damage induces the exposure of Mtb to cytosolic autophagy adaptors such as Optneurin, p62, NDP52 and NBR1 which bind to ubiquitin associated with Mtb as a consequence of the E3-ligases SMURF-1 and Parkin, culminating with targeting of the bacteria for autophagic degradation (center). Extracellular bacterial DNA from Mtb is detected by STING to activate TBK1 and lead to Mtb ubiquitination and recruitment of p62 and NDP52 (middle-left). IRGM-induced increase in ROS provokes autophagic targeting of Mtb (right).

In the last decade, autophagy emerged as an essential protective strategy employed by the host to restrict the spread of *M. tuberculosis*. The first piece of evidence on the role of autophagy in the control of *Mycobacterium* was provided by the cornerstone study of Gutierrez et al. (3). The authors demonstrated that upon the induction of autophagy by starvation or rapamycin *M. tuberculosis* variant *bovis* BCG colocalized to LC3^+^ compartments in RAW 264.7 macrophages. Moreover, BCG phagosomes were shown to be positive for markers of acidification such as cathepsin D and Lamp-1, suggesting that xenophagy induction was able to override the blockade in phagosome maturation by BCG, with a clear impact on bacterial killing (Figure 1). Interferon-γ (IFN-γ) is essential for resistance to infection, by interfering with the transcription of more than 2,000 genes (23). In a more physiological context, Gutierrez et al. demonstrated that IFN-γ, a potent activator of macrophages, was able to mimic the effects of rapamycin or starvation on the induction of autophagy, through the immunity-related p47 guanosine triphosphatases (IRG) Irgm1 (LRG-47) (3) (Figure 1). These results put autophagy on the center stage of the immune mechanisms involved in the protection against *M. tuberculosis* infection. After their initial discoveries, in a subsequent study, the same group demonstrated that both Irgm1 and its human ortholog IRGM are necessary for the induction of autophagy, generating large autolysosomes that contributed with *M. tuberculosis* intracellular growth restriction upon macrophage activation by IFN-γ (24). The mechanism behind IRGM restriction of *M. tuberculosis* seems to rely on its interaction with cardiolipin in mitochondria to generate ROS and mitochondrial fission, both necessary for *M. tuberculosis* killing (25). The role of IFN-γ in autophagy also involves the participation of interferon-induced guanylate-binding (GBP), which are also upregulated in the presence of the cytokine. It has been demonstrated that GBPs promote oxidative killing and the delivery of antimicrobial peptides to autophagolysosomes, contributing to *M. tuberculosis* intracellular replication control (26) (Figure 1). Altogether, these studies demonstrated an essential *in vitro* role for xenophagy and its induction by IFN-γ in the control of *M. tuberculosis* intracellular replication.

Although the link between deficiencies in ATG proteins and defective xenophagy has been widely reported upon infection with *M. tuberculosis*, ATG5 have also been described to play a critical autophagy-independent role in an *in vivo* TB mouse model. Kimmey et al. have demonstrated that the deletion of *Atg14L, Atg12, Atg16L1, Atg7*, and *Atg3* in the myeloid compartment did not affect the outcome of *M. tuberculosis* infection, suggesting that the loss of autophagy is not implicated with the progression of the disease. In sharp contrast, the authors reported that the loss of ATG5 in PMN but not in alveolar macrophages led to exacerbated imunopathology, sensitizing mice to *M. tuberculosis*. Together, these findings suggest that ATG5 has unique autophagy-independent features that are not shared with other ATG proteins, pointing for a reinterpretation of the role of ATG5 in the control of *M. tuberculosis* infection *in vivo* (27).

One primary open question that remained to be answered was how eukaryotic cells sense *M. tuberculosis* infection to induce autophagy. Toll-like receptors (TLRs) detect a myriad of extracellular and endolysosome located microbial products. It has been reported that Poly (I:C), LPS, and ssRNA, ligands for TLR3, TLR4, and TLR7, respectively, induce autophagosome formation through MyD88-dependent pathways. Interestingly, activation of TLR7 by its ligand increased the ability of macrophages to kill BCG (28) (Figure 1). However, it was not clear how TLRs would be able to detect BCG to induce autophagy in the absence of exogenous stimulation with their cognate ligands. The first molecular evidence of the detection of *M. tuberculosis*-derived microbial-associated molecular pattern (MAMP) triggering autophagy demonstrated that stimulation of interferon genes (STING), an important adaptor of TANK-binding kinase (TBK1) in the interferon stimulatory DNA pathway, senses the presence of cytosolic DNA to trigger the ubiquitination of *M. tuberculosis* after phagosome damage. Upon sensing of extracellular DNA from *M. tuberculosis* by STING, *M. tuberculosis* is ubiquitinated, leading to the recruitment by the autophagic adaptors p62/SQSTM1 (hereafter p62), a multi-domain protein that functions as an autophagic adaptor. p62 possesses an LC3-interacting protein region (LIR) and a C-terminal ubiquitin-associated (UBA) domain that binds ubiquitinated substrates and an LIR. Together with p62 and nuclear dot protein 52 kDa (NDP52) work to link ubiquitinated substrates to LC3 recruitment, ensuring the efficient delivery of *M. tuberculosis* to autophagosomes (29) (Figure 1). Although the sequestration of *Mycobacteria* by xenophagy has been demonstrated to be mostly dependent on ATG proteins, one report has been shown that sequestration of ubiquitinated mycobacteria can occur in ATG5-independent manner. The authors found that following 6-kDa early secretory antigenic target (ESAT-6) secretion system 1 (ESX-1)-mediated phagosome escape, ubiquitinated bacteria were resequestered by structures that resembled autophagosomes and localized to Lamp-1^+^ compartments. Notably, ubiquitinated *M. marinum* were never decorated with LC3 and ATG5 deficiency and did not affect bacterial counts. It remains to be elucidated if the finding that *M. marinum* did not localize to LC3^+^ compartments represents a potential specific mechanism of escape from autophagy (30).

Upon phagosome damage mediated by ESX-1, *M. tuberculosis* is ubiquitinated, in an essential step required for the recruitment of the autophagic adaptors p62 and NDP52 and LC3. Although it has not been determined, which bacterial or host proteins (or both) are ubiquitinated during xenophagy, much progress has been made in the identification of host proteins that mediate ubiquitination involved in xenophagy. Several ubiquitin-ligases have been described as participants of bacterial ubiquitination. Parkin has a well-established role in mitophagy where it promotes the ubiquitination of mitochondrial surface proteins prior to the recruitment of p62 in order to direct malfunctioning mitochondria for autophagic degradation. In 2013, Parkin was also reported to be crucial in the conjugation of K63-ubiquitin chains to *M. tuberculosis* inside macrophages. In line with this finding, *Park2*^−/−^ displayed increased *M. tuberculosis* replication in an *in vivo* TB model (31) (Figure 1). Of note, Parkin has also been demonstrated to participate in ubiquitination of other mycobacterial species such as *M. leprae* (32). Similarly, SMAD-specific E3 ubiquitin-ligase protein 1 (Smurf1) has been demonstrated to mediate K48- but not K63-ubiquitination and the recruitment of the autophagy adaptor NBR1 during *M. tuberculosis* infection to control its replication in human macrophages and to associate with bacteria present in the lung of patients with pulmonary TB (33). In their study, Franco et al. reported that Smurf1- but not Parkin-dependent ubiquitination is necessary for the recruitment of proteasome and NBR1 for the vicinity of *M. tuberculosis*. In contrast, K63 ubiquitination by Parkin but not Smurf1 is required for the recruitment of p62 to the bacterial surface (Figure 1). It remains to be elucidated why host cells employ different ubiquitin-ligases with apparent redundant roles for targeting *M. tuberculosis* for xenophagy. One possibility is that the apparent redundancy of Smurf-1 and Parkin is a countermeasure resulting from the ability of *M. tuberculosis* to escape from autophagy. Also, the different ubiquitin moieties added to *M. tuberculosis* surface could help in the recruitment of various adaptors. Indeed, Smurf1-mediated ubiquitination recruits the adaptor NBR1, which is not recruited by Parkin-mediated activity.

Several recent studies have reported that *M. tuberculosis* uses sophisticated mechanisms to escape xenophagy and replicate inside host cells. In addition to the induction of miR33 and miR33* expression to manipulate cellular metabolism and energy levels (34) and miRNA125a to inhibit UVRAG expression (35) (discussed later in this review), *M. tuberculosis* also induces the expression of other microRNAs (miRNAs) to circumvent xenophagy by interfering with different aspects of cellular physiology. This is the case of miR30A that has its expression increased during infection with *M. tuberculosis* to decrease Beclin 1 expression levels, leading to inhibition autophagosome elongation to promote intracellular survival of *M. tuberculosis* (36). Similarly, miR144* inhibits antimicrobial responses against *M. tuberculosis* in monocytes by targeting the expression of DNA damage-regulated autophagy modulator 2, allowing *M. tuberculosis* replication (37). In contrast, miR155 has been demonstrated to play a pro-autophagic role during *M. tuberculosis* infection. Wang et al. reported that miR155 targets Ras homolog enriched in brain (Rheb), a negative regulator of autophagy to accelerate the process of xenophagy. Inhibition of autophagy by *M. tuberculosis* seems to aim not only xenophagy but other essential steps of the immune response as well. It has been recently demonstrated that the bacterial factor PE_PGRS47 inhibits autophagy through an unknown mechanism to block MHC II antigen presentation and dampen adaptative immune responses against *M. tuberculosis* (38). Altogether, these studies provide compelling evidence that despite the crucial role of xenophagy as an antimycobacterial mechanism, *M. tuberculosis* has developed means to escape autophagy and replicate within macrophages.

#### Streptococcus pyogenes

**Figure 2:**
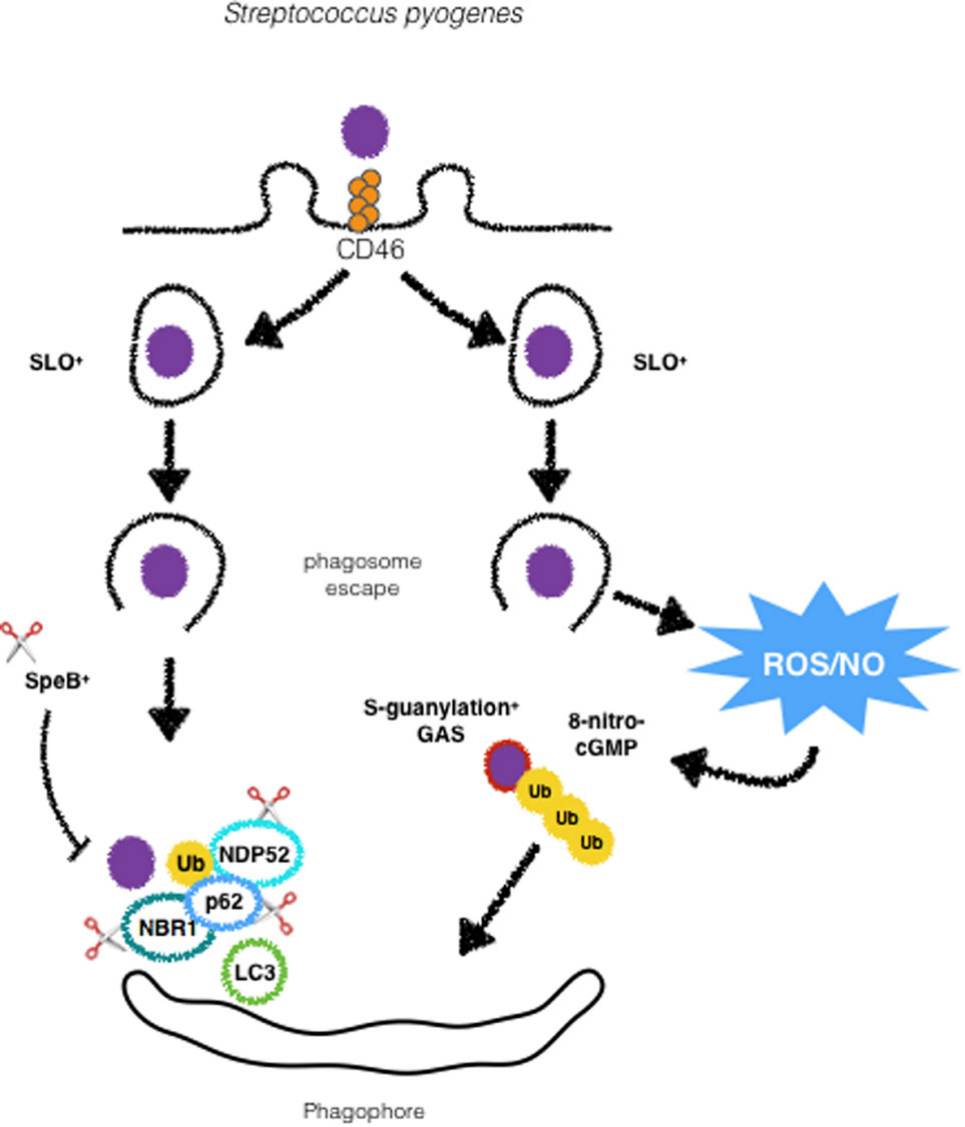
GAS is targeted by xenophagy by different mechanisms. Following activation of CD46, GAS is directed to autophagosomes. Streptolysin O promotes escape from phagosomes and ubiquitination and recognition by autophagic adaptors p62, NDP52 and NBR1. SpeB producing strains are able to degrade such adaptors to escape from xenophagy. GAS can also undergo modifications by ROS/NO-induced 8-nitro-cGMP via S-guanylation of its surface proteins followed by ubiquitination and targeting to autophagosomes.

*Streptococcus pyogenes* is the causative agent of a variety of infections, ranging from such as pharyngitis and skin infections to life-threatening necrotizin fasciitis and bacteremia (39). In 2004, Nakagawa et al. provide one of the first definitive evidence of the role of autophagy as a cell-autonomous antimicrobial mechanism. In this study, HeLa cells were shown to specifically target cytosolic GAS. This process was dependent on the toxin streptolysin O (SLO), a cholesterol-dependent pore-forming cytolysin (40). Nakagawa et al. demonstrated that the majority of the cytosolic population of GAS colocalized to LC3+ compartments, in contrast to SLO-deficient mutants in which no colocalization with LC3 was found (4) (Figure 2). As demonstrated for other intracellular bacteria, the adaptors p62, NDP52, and NBR1 are essential for recognition of ubiquitin decorated GAS and recruitment of LC3 before autophagic degradation (41, 42). Evasion of xenophagy by GAS has been reported, and GAS has been shown to evade ubiquitin recognition by the abovementioned autophagic adaptors. Barnett et al. have found that the globally disseminated serotype M1T1 (strain 5448) clone of GAS can avoid xenophagy to replicate in the cytosol. This is achieved by the expression of SpeB, a cysteine protease that degrades p62, NDP52, and NBR1. M1T1 Δ*speB* mutants fail to evade recognition by these proteins and are efficiently degraded through xenophagy (41) (Figure 2). These findings reveal a new mechanism by which GAS evades elimination by xenophagy. Notably, data from the literature demonstrate that xenophagy efficiently eliminates other GAS serotypes such as M6, M49, and M89. GAS is a successful human bacterial pathogen that causes a vast array of diseases and the work of Barnett et al. uncovers autophagy evasion as a determinant feature for the dissemination of GAS. The mechanisms employed by autophagy to target intracellular GAS also include the engagement of the CD46 pathogen receptor (43). CD46 is a glycoprotein expressed by all nucleated human cells that physically binds several pathogens such as adenoviruses B and D, human herpesvirus 6, *Neisseria*, and GAS (44) (Figure 2). Although several innate immune receptors such as TLRs have been described to trigger xenophagy upon infection or ligand stimulation, how these receptors are connected to the selective targeting of intracellular bacteria to lysosomes is still unclear. The findings from Joubert et al. provide an important piece of data to this open question. One possibility that needs to be experimentally tested is that CD46 might be concomitantly activated together with TLRs to promote xenophagy. Another known host factor that has been reported to participate in GAS targeting for xenophagy is 8-nitroguanosine 3’,5’-cyclic monophosphate (8-nitro-cGMP), a downstream mediator of nitric oxide that has been shown to promote protein *S*-guanylation on bacterial surface, which are then K63 ubiquitinated prior to the recruitment of LC3 (45). Although these findings shed light into a new xenophagy targeting mechanism during infection with GAS, some open questions remain, such as (i) is this mechanism specific for GAS? and (ii) which autophagy adaptors and ubiquitin-ligases participate in this process. One interesting question regarding the induction of xenophagy by GAS is the role of endothelial cells in this process. Despite different reports showing that xenophagy plays an important role in the clearance of intracellular GAS, in endothelial cells, the results are contrasting. While, Cutting et al. demonstrated the ability of endothelial cells to upregulate xenophagy in order to control GAS infection, a recent study from Lu et al. reports that endothelial cells fail to target GAS for degradation due to an intrinsic defect in the ubiquitination of intracellular bacteria (46, 47). Even though much progress has been done in the understanding of the mechanisms of GAS-induced autophagy, further studies are required in order to clarify whether endothelial cells are in fact defective in xenophagy, if this defect is specific for infection with GAS or if GAS can halt xenophagy in these cells and not in epithelial cells.

#### Shigella flexneri

**Figure 3:**
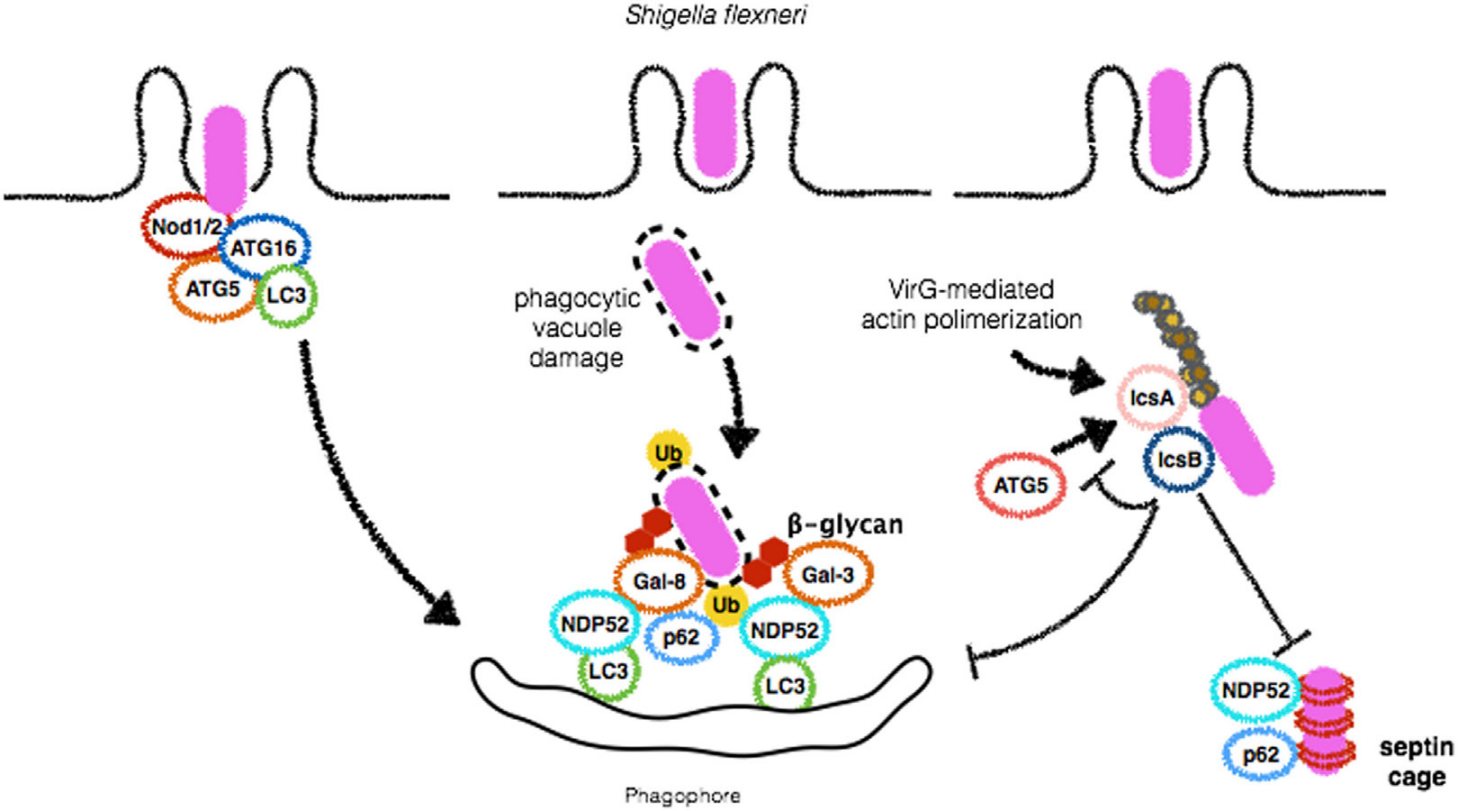
*Shigella flexneri* employs different mechanisms to escape from autophagy. During bacterial entry into host cells, Nod1 and Nod2 recruit ATG16L1 to initiate autophagosome formation in order to restrict *S. flexneri* replication (left). Vacuole damage leads to β-glycan exposure and recognition by Galectins 3 and 8 and recruitment of NDP52, followed by bacterial ubiquitination and binding to p62 and NDP52, culminating to *S. flexneri* targeting for autophagic degradation (center). IcsB plays a central role in disguising autophagic machinery. This protein competes with IcsA/VirG for binding to ATG5, preventing p62, and NDP52 binding, septin caging and autophagosome formation (right). Septin caging and further recruitment of autophagic adaptors are blocked by IcsB expression.

*Shigella* spp. are Gram-negative and highly invasive enteropathogens and a significant cause of disease, especially in children under the age of 5 years, causing approximately one million deaths worldwide (48). A few minutes after its invasion of epithelial cells and macrophages, *S. flexneri* is able to lyse the phagocytic vacuole and access the cytosolic compartment where it replicates (49). As countermeasures, host cells trigger autophagy to restrict *S. flexneri* intracellular growth and cell-to-cell spreading. The first evidence of an interaction between *Shigella* and autophagy was provided by a study dating from 2005. In this study, it was demonstrated that wild-type *S. flexneri* can escape from autophagic targeting by employing IcsB, one of the effectors of its type 3 secretion system (T3SS). Ogawa et al. observed that deletion mutants for IcsB, which is secreted by cytosolic bacteria and localizes to the bacterial surface were more efficiently targeted by autophagosomes. These results suggest that *S. flexneri* is able to escape from xenophagy. According to this study, the escape mechanism employed by *S. flexneri* involves IcsA/VirG, a 52 kDa protein that requires the bacterial chaperone IpgA for its stability, activates the complex-related proteins (Arp) 2/3 complex through the recruitment and activation of N-WASP, to induce actin polymerization and bacterial motility within the cell (50–54). Mechanistically, the study of Ogawa et al. demonstrated that, in Δ*icsB* mutants, IcsA/VirG triggers autophagy by binding to ATG5. According to the authors, IcsB inhibits IcsA/VirG affinity for ATG5. Thus, in wild-type *S. flexneri*, IcsB reduces IcsA/VirG affinity for ATG5 to initiate xenophagy (52) (Figure 3). More recently, a study added more complexity to the role of IcsB as a factor contributing to *S. flexneri* escape from autophagy. Baxt and Goldberg reported that IcsB also contributes to *S. flexneri* escape from xenophagy by recruiting transducer of CDC42-dependent actin assembly 1 (Toca-1) to prevent the recruitment of the adaptor NDP52 and LC3 (55).

The ubiquitination of *S. flexneri* has been reported to be essential for the recruitment of the adaptors p62 and NDP52 (56). However, in contrast to *S. typhimurium* and *M. tuberculosis* for which several ubiquitin-ligases that ubiquitinate bacterial surface have been described, the mechanism used to host cells to tag *S. flexneri* with ubiquitin is not clear. LUBAC is an ubiquitin-ligase that mediates the formation of M1-linked ubiquitin chains that culminate with xenophagy and bacterial degradation of *S. typhimurium* (see below). In contrast, LUBAC was reported to play no major role in the trafficking of *S. flexneri* to autophagosomes (57). According to this study, *S. flexneri* escapes from LUBAC-dependent ubiquitination by secreting the effector E3-ligase H1.4 to antagonize the activity of LUBAC (57). Despite the lack of substantial data on the how *S. flexneri* is ubiquitinated, different adaptors that bind ubiquitin are independently recruited to the bacterial surface. For instance, p62 and NDP52 have been demonstrated to be recruited to *S. flexneri* surface and to regulate xenophagy mediated by each other. In agreement with the notion of its anti-autophagic role, IcsB also contributed to *S. flexneri* escape from autophagy by hiding IcsA/VirG from ubiquitin coating (56) (Figure 3). The reason why *S. flexneri* recruits different adaptors *S. flexneri* is not clear. However, it is possible that p62 and NDP52 may recognize different ubiquitin linkages as a result of the activity of different ubiquitin-ligases. Another hypothesis is that their LIR domains could be able to recruit different LC3 homologs and different adaptors that could contribute to membrane recruitment from various sources for the formation of autophagosome around bacteria. These hypotheses still lack experimental confirmation.

Shortly after *S. flexneri* entry in epithelial cells, the phagocytic vacuole is ruptured, membrane remnants expose host sugars in the cytosol, and galectin 3 promotes ubiquitination and recruitment of p62 to support xenophagy (58). In contrast to other reports from the literature, the authors did not observe increased recruitment of p62 in Δ*icsB*.

Interestingly, members of the NF-κB pathway such as TRAF6 and NEMO and the peptidoglycan receptor Nod1 were reported to localize to these membrane remnants (58). Similarly, NLRP3, NLRC4, ASC, and Caspase-1 were also found associated with Shigella vacuolar membrane remnants. The physiological meaning of these findings is still to be defined. One possibility is that these membrane portions might be used for the activation of inflammatory cascades and that this process is likely to be regulated by autophagy. Another possibility is that by recruiting these proteins to its vicinity, *S. flexneri* modulates NF-κB activation and inflammation to favor its replication and spread.

Septins are conserved GTP-binding proteins that play critical roles in cell division, cytoskeletal dynamics, and membrane remodeling (59). These proteins have been shown to form cages around *S. flexneri* actively polymerizing actin. Interestingly, colocalization of septins, p62, and LC3 on *S. flexneri* bacterial surface has been demonstrated and depletion of septins markedly reduced xenophagy of *S. flexneri*, suggesting an intimate relationship between these two processes (60). More recently, the precise mechanisms involved in *S. flexneri*-cage assembly were revealed. Sirianni et al. have found that mitochondrial proteins associate with *S. flexneri*-septin cages and that mitochondria promote the formation of septin cage assembly around *S. flexneri* for antibacterial xenophagy (61). *S. flexneri* has been demonstrated to induce mitochondrial damage and in the study by Siriani et al., this aspect was linked to dampening of septin cages and escape (61, 62). Of note, IcsB contributes to masking *S. flexneri* from septin caging (60). These results demonstrate that IcsB dampens xenophagy by at least three different mechanisms: competing with IcsA/VirG for binding to ATG5, by avoiding septin caging and targeting to autophagosomes, and by recruiting Toca-1 to inhibit the recruitment of NDP52 and LC3.

In addition to direct interaction of its virulence factors and autophagy proteins, pattern-recognition receptors also seem to participate in the interplay between *S. flexneri* and autophagic pathways. It has been demonstrated that the infection of macrophages by *S. flexneri* induces a robust activation of Caspase-1 that leads to inflammasome activation and cell death by pyroptosis in an NLRC4-dependent but ASC-independent mechanism (63). Interestingly, both Caspase-1 and NLRC4 were shown to negatively regulate autophagosome formation in macrophages infected with *S. flexneri* as demonstrated by studies in which bone marrow-derived macrophages (BMDMs) from knockout mice for the genes encoding these proteins were shown to induce the formation of GFP-LC3 positive membranes around bacteria in contrast to wild-type BMDMs (63). In contrast to previous studies, IcsA/VirG was not implicated in autophagy induction (52, 63), which can be explained by the different cell types used in these studies. In contrast to negative regulation of autophagy by NLRC4, NLRC1 (Nod1), and NLRC2 (Nod2), the founding members of the NLR family have been linked to autophagy induction. Nod1 and Nod2 are sensors of intracellular peptidoglycan that upon engagement lead to the activation of NF-κB activation through the recruitment of the adaptor protein RIP2 (64). Both Nod1 and Nod2 have been shown to recruit ATG16L1 at early stages of infection by *S. flexneri* to initiate autophagosome formation. As a result, Nod1- and Nod2-deficient MEFs display decreased numbers of GFP-LC3 positive bacteria, and interestingly, these findings did not rely on recruitment of RIP2 or NF-κB activation. Notably, in this study, the most common Nod2 mutation associated with Crohn disease (CD) resulted in impaired recruitment of ATG16L1 to the bacterial entry site and much less xenophagy, underscoring the notion that dysregulation of bacterial autophagy is likely to play an important role in the pathogenesis of CD (49) (Figure 3). It remains to be clarified if and in which conditions Nod1/2-dependent pro-autophagic signals would prevail over NLRC4-dependent anti-autophagy ones and *vice versa*.

#### Salmonella typhimurium

**Figure 4:**
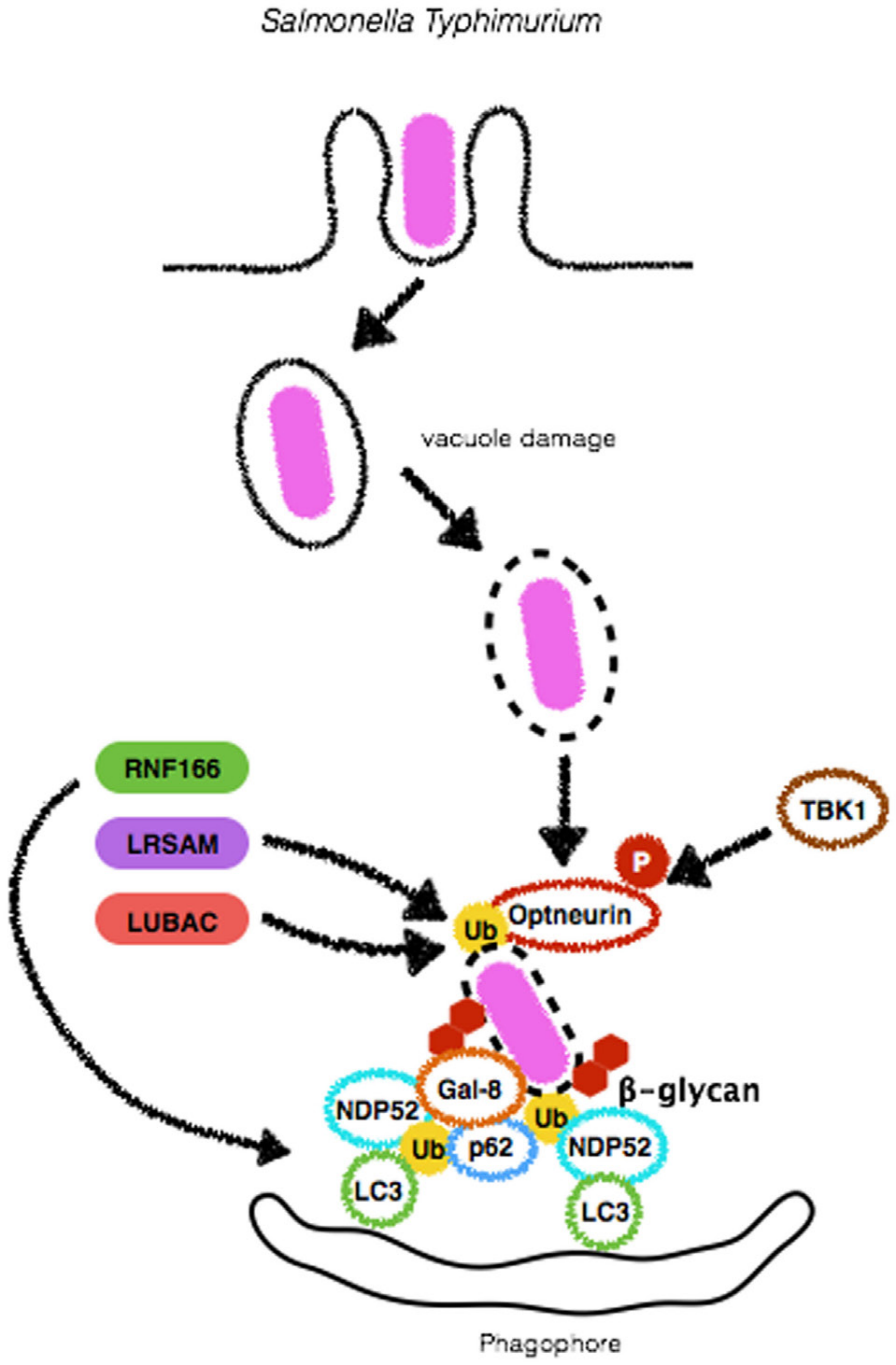
Mechanisms of autophagy induction by *Salmonella Typhimurium*. Upon entry in epithelial cells, *S. Typhimurium* resides in a specialized compartment, the Salmonella-containing vacuole (SCV). A fraction of the bacterial population damages and/or escapes from SCV and initiates to replicate in the cytosol. Either still within the SCV or free in the cytosolic compartment, *S. Typhimurium* triggers autophagy by several means. β-glycan present in vacuole remnants is recognized by Galectin-8 (Gal-8) and targets bacteria to autophagosomes. *S. Typhimurium* can also be ubiquitinated by the E3-ligases LRSAM or LUBAC, allowing its recognition by autophagic adaptors Optneurin, p62 or NDP52. RNF166, another E3-ligase, ubiquitinates p62 to increase the ability of this protein to bind bacteria-associated ubiquitin. Diacylglycerol (DAG) recognition and autophagy induction upon *S. Typhimurium* infection are not depicted here.

*Salmonella typhimurium* is a pathogenic Gram-negative bacterium found in the intestinal lumen and a major cause of gastroenteritis in humans and other mammals (65). This pathogen uses two T3SS, encoded by *Salmonella* pathogenicity island 1 and 2 (SPI2) to enter non-phagocytic cells and establish a replicative niche within vacuoles termed *Salmonella*-containing vacuole (SCV). In 2006, it was first reported that a fraction of the bacterial population within the SCV previously demonstrated to form pores in this compartment was able to reach the cytosol being immediately targeted by LC3 and ATG proteins. In this study, the authors showed that *Atg5*-deficient MEFs infected with *S. typhimurium* had decreased fusion of LC3^+^ bacteria colocalized with Lamp1, suggesting diminished bacterial degradation in lysosomes. Indeed, these cells harbored increased bacterial numbers, confirming the role of autophagy in the control of *S. typhimurium* infection (66) (Figure 4). Importantly, xenophagy has been reported to be essential in the control of *S. typhimurium* in other models such as *Caenorhabditis elegans* and *Dictyostelium discoideum*, suggesting that the role of xenophagy as an anti-*S. typhimurium* mechanism has been conserved throughout evolution (67).

Although *S. typhimurium* targeting by autophagy was demonstrated, the means by which autophagosome formation machinery recognizes cytosolic *Salmonella* is not completely clear and has been the subject of many studies. Cytosolic, but not SCV residing bacteria, have been shown to be decorated with ubiquitin early during infection (66, 68). Interestingly, Ub^+^ bacteria colocalize with LC3, suggesting that the autophagic machinery can detect ubiquitinated substrates. Indeed, p62 has been shown to play a crucial role in the recognition, targeting to lysosomes and restriction of cytosolic ubiquitinated *S. typhimurium* (69). Other ubiquitin-binding proteins have also been reported to participate in *Salmonella*-induced autophagy. Similarly to p62, Optineurin harbors LIR and UBA domains and was shown to be necessary for the control of *S. typhimurium*. Interestingly, this mechanism requires Optineurin to be phosphorylated by TBK1 on serine-177 in order to enhance ubiquitin- and LC3-binding affinity to promote bacterial clearance (70). One aspect of *S. typhimurium* recognition by autophagy machinery that remained elusive was which bacterial substrates are ubiquitinated prior to detection by the adaptors p62, NDP52, and Optineurin. A recent study from Fiskin et al. in which ubiquitination site profiling was performed during infection with *S. typhimurium* revealed that outer membrane proteins are targets for ubiquitination (71).

Several ubiquitin-ligases have been reported as necessary for ubiquitination of *S. typhimurium*. Leucine-rich repeat and sterile α-motif-containing 1 (LRSAM1) was shown to play an essential role in the autophagic degradation of *S. typhimurium*. This E3-ligase was found to localize to cytosolic *Salmonella* upon infection of epithelial cells to ensure proper ubiquitination and autophagic control of bacterial replication. In line with these findings, a cohort study reported that lymphoblasts from patients with Charcot-Marie-Tooth disease, which harbor a frameshift mutation that truncates the RING domain of LRSAM1, present limited antibacterial activity as compared to cells from control individuals (72, 73). Another E3-ligase demonstrated to be involved in autophagic targeting of *S. typhimurium* is RNF166. This gene was identified in a screening for human E3-ligases as necessary for the recruitment of p62, NDP52, and LC3 for the bacterial surface in order to limit *S. typhimurium* replication. A unique feature of RNF166 is that, rather than tagging bacteria, it drives K29- and K33-linked ubiquitination of p62 at K91 and K189. According to the authors, this step is essential for p62-dependent bacterial targeting for autophagosomes (74) (Figure 4). More recently, the role of LUBAC, another E3-ligase, has been described. LUBAC generates linear (M1-linked) polyubiquitin patches on the surface of *S. typhimurium* to recruit the adaptors Optineurin, NDP52 and p62 and direct bacteria for autophagic degradation. Indeed, MEFs from *cpdm*^−/−^ mice, which harbor a spontaneous mutation in LUBAC or MEFs silenced for the protein, display an increased time-dependent replication of *S. typhimurium* in comparison to wild-type or control-silenced cells, respectively (57). In addition to the recruitment of autophagy adaptors, LUBAC was reported to be crucial in triggering pro-inflammatory roles during infection with *S. typhimurium* (Figure 4). LUBAC-dependent generation of M1-linked polyubiquitin chains on the surface of the bacteria also recruits NEMO to this site (57, 75). These findings are of particular interest as it suggests that bacterial surface can provide mechanical support for the assembly of signaling platforms such as NF-κB activation, a major transcription factor that controls the production of inflammatory mediators such as cytokines and chemokines. Given that LRSAM1 was found to be only partially responsible for *S. typhimurium* ubiquitination, which RNF166 ubiquitinates p62 rather than bacteria and that LUBAC required an upstream E3-ligase, Polajnar et al. hypothesized that other ubiquitin ligases were involved in the ubiquitination of *S. typhimurium* and identified Ring-between-Ring E3 ligase ARIH1 (also known as HHARI) as an important protein ubiquitin-ligase for targeting this pathogen to autophagosomes (76). Notably, this study demonstrated that depletion of LRSAM1 and ARIH1 led to an enhancement in LUBAC-dependent ubiquitination and NF-κB activation, culminating with increased bacterial replication, in contrast to previous findings, reporting that NF-κB activation led to bacterial growth restriction (57, 75, 76). Together, these data indicate that recruitment of different ubiquitin-ligases (with different ubiquitin linkage abilities) to the bacterial surface may endow cells with several layers of protection against the replication of cytosolic *S. typhimurium*.

In addition to bacterial ubiquitination, lipid second messengers have also been reported to be required for efficient targeting of *S. typhimurium*. Shahnazari et al. demonstrated that diacylglycerol (DAG) is produced during infection with *S. typhimurium* in a phospholipase D- and phosphatidic acid phosphatase-dependent manner. DAG localization in bacteria-containing phagosomes seemed to be a requisite for autophagy and may occur in parallel to independent p62 and NDP52 recruitment, once again suggesting several layers of proteins involved in bacterial targeting (77).

The detection of damage in the SCV has been demonstrated to be an important step in the targeting of *S. typhimurium* for autophagic degradation (66). Galectin-8 is a β-galactoside-binding lectin that has been reported to monitor endosomal and lysosomal integrity and detects bacterial invasion by binding host glycans exposed on damaged SCVs. Recently, it has been demonstrated that among galectins 1–4, 7–10, and 12–14, only Galectin-8 colocalized to *S. typhimurium* during infection of HeLa cells. Interestingly, NDP52 was recruited to cytosolic exposed *S. typhimurium*, directly binding to Galectin-8 to restrict bacterial replication. These and previous findings lead to a model in which, upon SCV damage, host sugar molecules such as β-galactoside, usually confined to the lumen of endosomes are exposed in the cytosol and sensed by Galectin-8 that in turn recruits NDP52 and LC3 to SCV to initiate lysosomal degradation of *S. typhimurium* (42, 78). Despite their role in mediating *S. typhimurium*-induced autophagic degradation, p62 and NDP52 show independent targeting activity. In a study in which HeLa cells were silenced for p62 or NDP52, there was no interference in the number of NDP52^+^ or p62^+^ bacteria, respectively (Figure 4). Interestingly, it was demonstrated that these adaptors recognize ubiquitin deposited in distinct microdomains at the bacterial surface that could result from the activity from different ubiquitin-ligases (79). Future studies must provide explanations if and why cells preferably decide toward the employment of one or the other ubiquitin-ligase and autophagy adaptors.

#### Listeria monocytogenes

*Listeria monocytogenes* is a Gram-positive bacterial pathogen that causes listeriosis, a self-limiting disease in healthy individuals that become severe in immunocompromised or elderly individuals and pregnant women (80). One of the main features of *L. monocytogenes* is its ability to replicate within several cell types during infection, including macrophages, a cell type usually able to kill the majority of intracellular bacteria (81).

Before its replication in the cytosol, *L. monocytogenes* must escape from the phagosome. This is achieved through the expression of several virulence factors rapidly upon entry. Possibly, the main bacterial factor associated with phagosome escape, listeriolysin O (LLO), is a cholesterol-dependent, pore-forming cytolysin that form pores in the phagosomal membrane immediately after bacteria uptake (82–85). In experiments with fluorescently labeled molecules of increasing sizes, it has been demonstrated that the pores grow in size until large enough to allow bacterial escape (86). In addition to LLO pore-forming activity, two C-type phospholipases, phosphatidylinositol-specific (PI-PLC, plcB), and a broad-range phosphatidylcholine (PC-PLC, plcA) also contribute to *L. monocytogenes* escape from phagosome, likely digesting its membrane (86).

**Figure 5:**
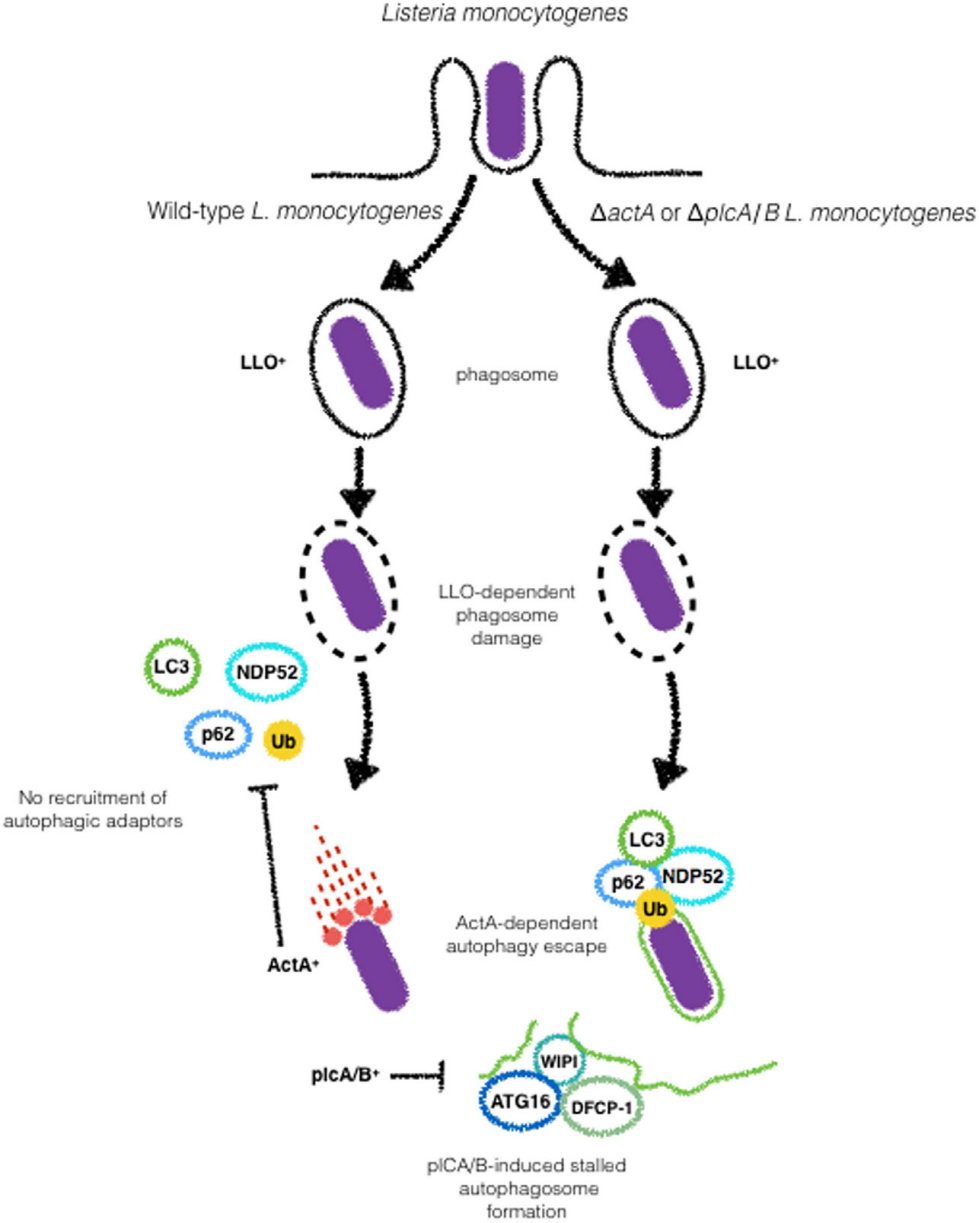
ActA and phospholipases (plc) A and B are major *Listeria monocytogenes* virulence factors involved in the escape from autophagy. Upon Listeriolysin O-dependent escape from phagosome wild-type *L. monocytogenes* escapes from autophagic targeting due to the expression of ActA, that blocks the recruitment of p62 and NDP52 to the bacterial surface. The expression of plcA/B stalls the formation of autophagosomal membranes. Galectin recognition of damaged vacuoles and autophagy induction are not shown here.

In order to successfully replicate in the cytosol, *L. monocytogenes* needs to circumvent several layers of host defense. Autophagy has been reported to contribute to the control of infection, although several studies show that the bacteria are able to escape from autophagic degradation (87, 88). Infection of RAW 264.7 macrophages of wild-type *L. monocytogenes* showed that ~40% of the intracellular bacterial population was targeted by LC3 by 1 h postinfection (p.i) in an LLO-dependent manner (Figure 5). However, at 8 h p.i, only 10% of the bacterial population was LC3-positive. These results suggest that *L. monocytogenes* was able to escape from autophagic degradation. Indeed, after initial targeting by LC3, replication rates robustly increased, in line with the drop in bacterial colocalization with LC3 observed at later stages of infection (89).

ActA, a key virulence factor of *L. monocytogenes* involved in intracellular motility, has also been implicated in autophagy evasion. *In vitro* studies demonstrate contradictory results with Δ*actA* mutants in different genetic backgrounds and cell types. While EGDe Δ*actA* mutants infecting Hela cells show time-dependent increase in the colocalization with LC3, 10403S Δ*actA* mutants in the 10403S background infecting macrophages loses its staining for LC3 at later time points during infection (88, 90). It remains to be elucidated whether the differences observed for both genetic backgrounds are related or not to the different cell types used. Despite this controversy, it is important to note that Δ*actA* mutants in both backgrounds display comparable replication *in vitro* (88–90). Importantly, ActA-dependent escape of autophagy does not rely on its ability to mediate bacterial motility. Using a series of *ActA* truncated mutants, Yoshikawa et al. demonstrated that as long as the capacity of ActA to recruit actin-related proteins (Arp) 2/3 complex, vasodilator-stimulated phosphoprotein or actin, is retained, bacteria are able to disguise autophagic recognition (88) (Figure 5).

The ubiquitination of *L. monocytogenes* and the involvement of autophagy adaptors such as p62 and NDP52 have been reported in the targeting *L. monocytogenes* to autophagosomes. In HeLa cells, p62 and NDP52 were shown to be recruited independently during the infection with the Δ*actA* EGDe (56). Recently, these strains were compared in regards to LC3, p62, and Ub during infection of macrophages. Although Δ*actA* mutants in EGDe and 10403S genetic backgrounds were reported to be sharply different regarding colocalization with LC3, p62, and Ub recruitment and replication were identical for both strains suggesting that Δ*actA* can block xenophagy downstream of ubiquitination and LC3 recruitment (90). The ubiquitin-ligases Parkin and SMURF1 were demonstrated to play a role in the ubiquitination of *L. monocytogenes*. *Park2*^−/−^ mice infected with *L. monocytogenes* showed up to 20-fold higher bacterial load relative to wild-type animals (31). *Smurf1*^−/−^ macrophages infected with Δ*actA L. monocytogenes* do not show recruitment of K48-ubiquitin to the bacterial surface while K63-linked ubiquitination was not affected. In line with this finding, *Smurf1*^−/−^ mice infected with *L. monocytogenes* were shown to harbor significantly higher bacterial burdens in comparison to wild-type (33). As ubiquitination of cytosolic bacteria has been known to be essential for autophagic degradation, it is assumed that the higher bacterial burden in *Smurf1*^−/−^ mice is a consequence of dampened antibacterial autophagy. More recently, NEDD4 (neuronal precursor cell expressed, developmentally downregulated 4), another ubiquitin-ligase has been implicated autophagic degradation of *L. monocytogenes* (Figure 5). However, in contrast to Parkin and Smurf1, NEDD4 does not recruit ubiquitin to the bacterial surface but enhances the mediated K6- and K27-linkage ubiquitination of BECN1, leading to higher stability of BECN1 and increased autophagy (91).

*Listeria monocytogenes* has been reported to induce amino acid starvation and activation of the general control nonderepressible 2 (GCN2)-eIF2α pathway upstream mTOR. GCN2 is one of four “stress kinases” that block translation by phosphorylating eIF2α. GCN2 is thought to bind uncharged tRNAs to “sense” amino acids availability (92). Upon detection of a decrease in the amino acid pool, mTOR activity is reduced leading to autophagy activation to normalize this condition. Unlike what is observed during the infection of epithelial cells with *S. flexneri*, in *L. monocytogenes-infected* cells, autophagy is kept repressed, suggesting that *L. monocytogenes* possesses other virulence weapons to block autophagy (93–95). In addition to Δ*actA-*mediated escape from autophagy, *L. monocytogenes* employs its two C-type phospholipases to disrupt the autophagosome elongation step in order to inhibit autophagy-dependent degradation. In an *in vitro* study, it was observed that *L. monocytogenes* deleted for plcA and plcB were more strongly targeted to autophagosomes than wild-type bacteria at later time points of infection. In parallel, wild-type bacteria induced the accumulation of granules positive for LC3, ATG16L1, and as well as WIPI-2, a phosphatidylinositol 3-phosphate-binding protein that is present on maturing phagophores, suggesting blockade of pre-autophagosome structures. Interestingly, the authors demonstrate that in plcA/plcB *L. monocytogenes* mutants, the accumulation of such structures was not observed (93, 95). These results, together with the previous findings of Mitchell et al. point toward the combined effects of ActA and *L. monocytogenes* phospholipases in the escape from autophagy (87) (Figure 4).

The detection of MAMPs has also been described as an autophagy trigger during the infection of *L. monocytogenes*. In 2008, a study using *Drosophila melanogaster* as a model for *L. monocytogenes* infection reported that peptidoglycan-recognition protein (PGRP-LE) mediated autophagy-dependent control of bacterial replication *in vitro* and *in vivo* (96). Interestingly, the intracellular peptidoglycan receptor Nod1 has also been linked to xenophagy of *L. monocytogenes in vitro*. MEFs from Nod1-deficient mice were demonstrated to be defective in targeting *L. monocytogenes* to autophagosomes, indicating an important role for peptidoglycan recognition in the induction of autophagy during infection with this bacterium in mammals as well (49).

#### Legionella pneumophila

**Figure 6:**
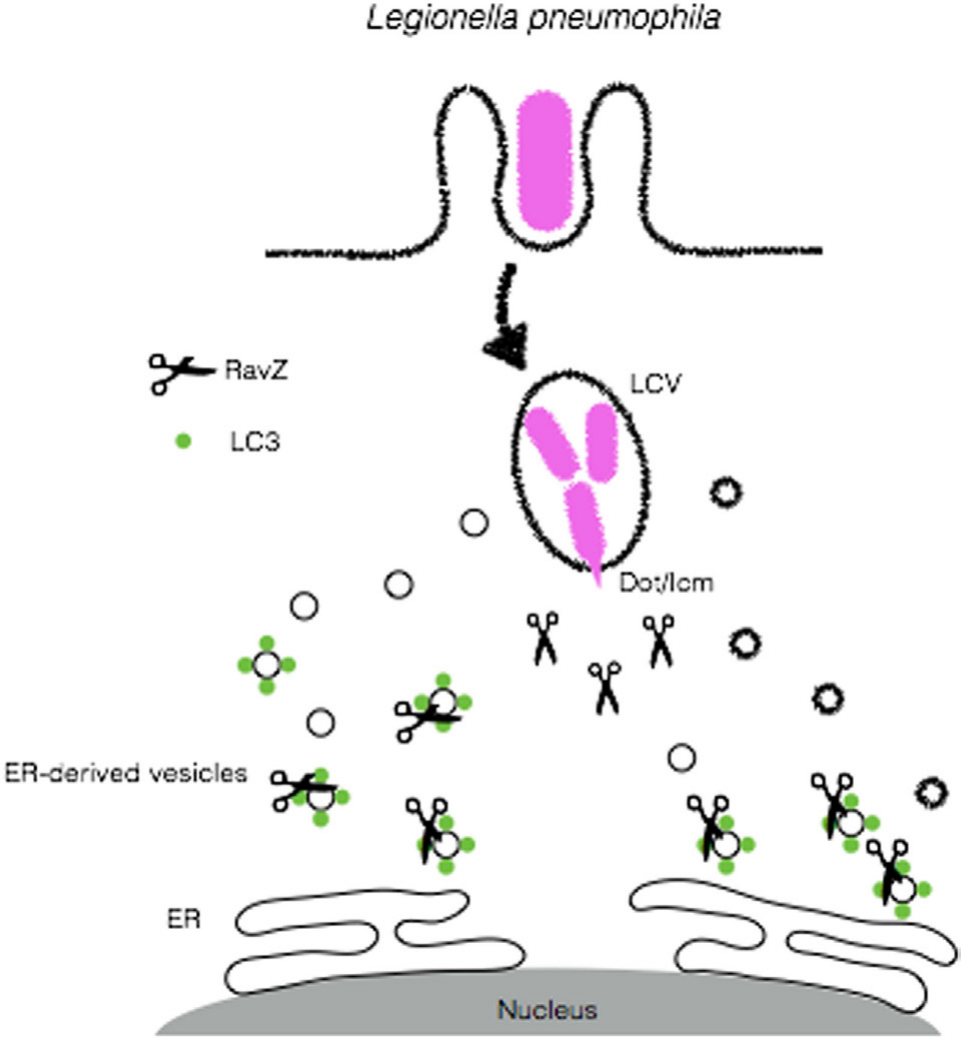
*Legionella pneumophila* disrupts autophagy to create a replicative niche. *L. pneumophila* secretes RavZ through its Dot/Icm apparatus in order to deconjugate LC3 from ER-derived vesicles and block autophagy in order to escape from autophagic degradation.

The Gram-negative bacterium *L. pneumophila* was first identified as the causative agent of an epidemic of pneumonia at an American Legion convention in Philadelphia, PA, USA in 1976 (97). This disease is characterized by the inhalation of aerosols containing high numbers of *L. pneumophila* (98). Although usually found in freshwater protozoa and amebae, *L. pneumophila* can accidentally replicate in alveolar macrophages in human lung, especially in immune-compromised patients (99, 100). In order to replicate within its eukaryotic host, *L. pneumophila* employs strategies that involve blocking the fusion of phagosomes with lysosomes after phagocytic ingestion of the bacteria and the generation of endoplasmic reticulum (ER)-like compartment that affords its replication (7, 101). The *L. pneumophila*-containing vacuoles (LCVs) present features that are shared by autophagosomes, including its close association with ER membrane (100, 102). This led to the speculation that the formation of biogenesis could involve the autophagy machinery (103, 104). Initial studies that focused on the characterization of the LCV reported that this compartment did not fuse with acidic vesicles since proteins that localize to endolysosomes, such as Lamp-1 and Rab7 were absent in LCV membrane and that the ability of *L. pneumophila* to evade phagosomal maturation was dependent on its viability (98, 105). Further studies using avirulent strains of *L. pneumophila* identified the intracellular multiplication (icm) and defect in organelle trafficking (dot) loci as the genetic loci determinants required for intracellular multiplication and evasion phagosome–lysosome fusion (106, 107). The emergence of autophagy as an antimicrobial effector led to the examination of the role of this process in the pathogenesis of *L. pneumophila* infection. Since LC3 is a major marker for autophagosome membranes, several cell biology approaches aimed to analyze the recruitment of LC3^+^ compartments to LCVs (100). Interestingly, following infection of macrophages with *L. pneumophila*, the formation of autophagosomes was blunted. In line with the role of Dot/Icm in the virulence of this bacterium, infection of macrophages with an isogenic Dot/Icm-deficient dotA mutant was unable to induce defects in autophagy induction (108). To identify the bacterial factors involved in autophagy inhibition, Choy et al. conducted a genetic screen that mapped the defect in autophagy to a chromosomal region encoding for 10 effectors. Analysis of the effects of the individual effectors revealed the protein RavZ as necessary and sufficient for blocking autophagy (108). *In vitro* analysis demonstrated that RavZ, which displays cysteine-protease activity, acts to deconjugate LC3 from autophagosomes and block its reconjugation (108). Furthermore, recent reports demonstrate that RavZ might participate not only in the deconjugation of LC3 but also in other steps that interfere with xenophagy. Kubori et al. have found in co-infection experiments with *L. pneumophila* and *S. typhimurium* that the recruitment of ubiquitin, p62, and NDP52 to the surface of *S. typhimurium* was dampened, suggesting a deubiquitinase-like enzymatic activity for RavZ (109). The resolution of the crystal structure of RavZ yielded new clues to its mechanisms. According to this study, by targeting autophagosomes through PIP3- and curvature-sensing motifs, RavZ limits its activity only to LC3 that is bound to autophagosomes (110) (Figure 6). Other RavZ-independent mechanisms for *L. pneumophila* evasion of autophagy have been described as well. Phylogenetic analyses suggested a high degree of similarity between one *L. pneumophila* and the eukaryotic sphingosine-1 phosphate lyase (SPL) (111). The *L. pneumophila* SPL homolog (LpSlp) has similar enzymatic activities to the eukaryotic SPL, which finely regulates intracellular levels of sphingosine-1-phosphate (S1P) (112), which have been shown to stimulate autophagy (111, 113). Infection of macrophages with wild-type *L. pneumophila* but not the LpSpl-deficient mutant leads to a depletion in S1P levels and inhibition of autophagy, indicating that *L. pneumophila* uses molecular mimicry to block autophagy and replicate within macrophages (111).

#### The Role AMP-Dependent Protein Kinase (AMPK) Activation and Bacterial-Induced Amino Acid Starvation in Bacterial Xenophagy

Living organisms obtain energy from the catabolism of nutrients whose molecular blocks are then converted into ATP and NADPH. The fact that cells are continually synthesizing ATP keeps its level close to maximal, with only small variations (114). However, under nutrient stress, when ATP levels drop, adenylate kinase shifts to an ATP synthesis mode to restore its levels. In turn, AMP levels increase significantly and, physiologically, changes in AMP concentrations are much higher than those observed to ATP (115), which makes the AMP/ATP ratio the most reliable marker of the cellular energetic status (114). Under such conditions, AMPK detects tiny changes in AMP levels and represents the principal cellular metabolism regulator (114). One of the main direct consequences of AMPK engagement is the activation of ULK1, suppressing mTORC1 inhibitory activity to allow the formation of autophagosomes (116).

In addition to its crucial role as a metabolic sensor, AMPK has also been widely reported to be involved in the activation of autophagy by bacteria. In a bacterial peritonitis-induced sepsis model, the use of the AMPK activator aminoimidazole carboxamide ribonucleotide (AICAR) increased bacterial killing, suggesting the implication of AMPK in the enhancement of the activity of phagocytic cells. Indeed, the use of these activators led to increased chemotaxis, phagocytosis, and bacterial killing of neutrophils infected with *Escherichia coli* (117). Evidence from the literature demonstrates that activation of AMPK by treatment with AICAR can also increase targeting of *M. tuberculosis* to LC3-positive compartments. Of note, when key autophagic proteins such as ATG7 were silenced, this effect was not observed, suggesting AICAR promotes the targeting of *M. tuberculosis* to autophagosomes. Moreover, AICAR-induced xenophagy was shown to contribute to bacterial killing *in vitro*, in a mechanism involving mTOR inhibition and increased mitochondrial biogenesis and ATP generation, likely as a result of energy drop during *M. tuberculosis* infection. Since it has been previously demonstrated that peroxisome proliferator-activated receptor-gamma, coactivator 1α (PPARGC1A) is important for the regulation of mitochondrial gene expression and glucose metabolism, it was speculated that PPARGC1A was involved in AICAR-induced xenophagy of *M. tuberculosis*. When PPARGC1A expression was silenced in macrophages infected with *M. tuberculosis*, the robust increase in mitochondrial biogenesis, ATP generation, and decreased *M. tuberculosis* replication induced by AICAR treatment were not observed (118, 119). The findings of this study support those from Gutierrez et al. (3) demonstrating that induction of autophagy through rapamycin enhances antimicrobial defenses against *M. tuberculosis*. Although AMPK activation was found to be involved in the efficient xenophagy-dependent control of *M. tuberculosis*, this pathogen developed sophisticated mechanisms to manipulate AMPK activity in order to favor its replication. In another recent evidence, miRNAs emerged as important “fine-tuners” of gene expression in response to pathophysiological stimuli. These RNAs bind to the 3’-untranslated region of specific mRNAs to reduce protein expression by blocking mRNA translation or inducing its degradation (120). Accumulating evidence shows that many miRNAs regulate the complex interplay between mycobacterial survival strategies and host innate immune and metabolic pathways (121). One of these miRNAs, miR33 has been shown to the regulation of fatty acid metabolism and insulin signaling (122). *M. tuberculosis* seems to use the expression of miRNAs to subvert autophagy to create a favorable replicative niche. *M. tuberculosis* infection of macrophages induces the expression of miR-33 and its passenger strand miR-33* to dampen mitochondrial fatty acid oxidation and lipophagy (autophagy of lipid droplets) to increase cellular lipid content, which is essential for the bacilli as a nutrient source during infection (34). According to this study, autophagy inhibition was achieved by inhibition of AMPK, which controls transcription factor EB and Forkhead box transcription factor class O (FOXO3), transcriptional regulators of autophagy and lysosomal biogenesis gene programs, respectively (123, 124). Altogether, these studies demonstrate that AMPK is activated during infection with intracellular bacteria.

**Figure 7:**
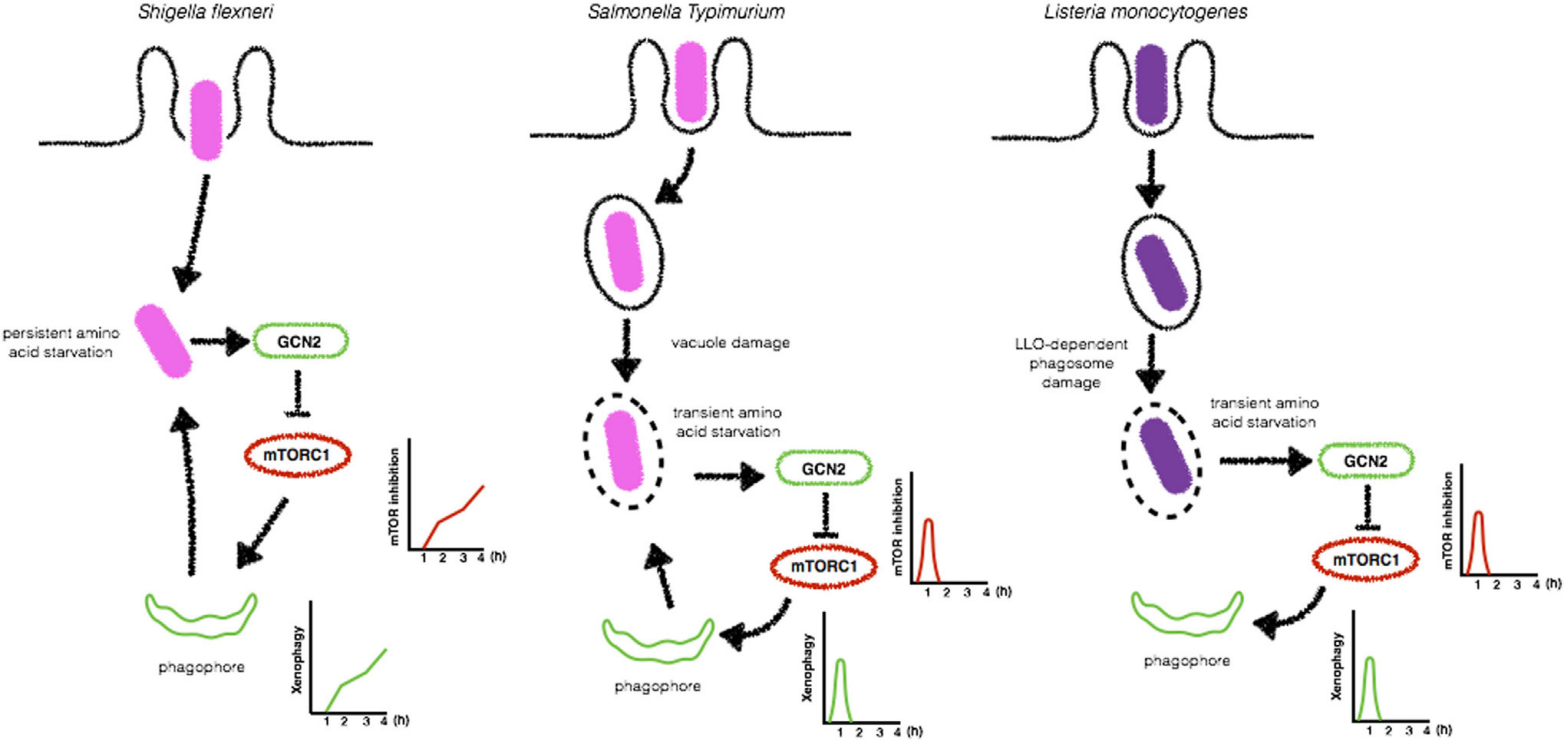
Bacteria-induced amino acid starvation stress triggers xenophagy. *Shigella flexneri* induces a persistent amino acid starvation that leads to GCN2 activation and inhibition of mTORC1 to allow the formation of bacteria targeted autophagosomes (left). In contrast, *Salmonella Typhimurium* (center) and *Listeria monocytogenes* (right) trigger only a transient amino acid starvation and inhibition of mTORC1, allowing its reactivation at later time-points to block the formation of autophagosomes.

The mechanisms by which intracellular bacteria initiate xenophagy are not completely elucidated, but compelling evidence from the literature suggests that these pathogens trigger energy imbalance and cellular nutritional stress that result in the activation of cellular responses culminating in the upregulation of autophagic activity (93). It has been reported that the infection of epithelial cells with *S. flexneri* infection induces a general and persistent loss of amino acids, leading to amino acid starvation-induced stress (94). In contrast, *S. typhimurium* induces only a rapid and transient depletion of amino acid pools. Of note, during *S. flexneri*- and *S. typhimurium*-induced amino acid depletion, robust relocalization of mTORC1 is observed. While in *S. flexneri*-infected cells, S6K1 and 4EBP1, two major targets of mTOR, are downregulated, and mTORC1 dispersed in the cytosol throughout infection, during the infection with *S. typhimurium*, this is observed only in early time-points, suggesting that this bacterial pathogen developed means to manipulate mTOR signaling to favor its intracellular survival (94) (Figure 7). Indeed, in a recent study, Ganesan et al. demonstrated that despite sustained low levels of ATP in macrophages infected by *S. typhimurium*, AMPK was only transiently activated at early time-points and then returned to basal levels (125). AMPK activation is known to be regulated by a cytosolic complex consisting of liver kinase B1 (LKB1) and Sirtuin-1 (Sirt1), where Sirt1 is necessary for deacetylation and activation of LKB1 (126). Interestingly, the study by Ganesan et al. reports that *S. typhimurium* induces the lysosomal degradation of AMPK, LKB1, and Sirt1 to reactivate mTORC1 activity in order to inhibit autophagosome formation and escape from xenophagy. Notably, this reactivation was shown to be dependent on SsrB, a regulator of pathogenicity island 2 (SPI2) encoded virulence factors (127), and SsaV a component of the SPI2 type III secretion apparatus (128). *S. typhimurium* mutants lacking SsrB and SsaV failed to induce lysosomal degradation of the AMPK/LKB1/Sirt1 circuit and are efficiently targeted to autophagosomes (125). Together, the studies from Tattoli et al. and Ganesan et al. demonstrate that nutritional cellular stress induced by bacterial infection triggers xenophagy to control bacterial replication (in the case of *S. flexneri* infection) and that *S. typhimurium* developed mechanisms to escape from autophagy by reactivating mTORC1 activity. Together, the studies from Tattoli et al. and Ganesan et al. demonstrate that nutritional cellular stress induced by bacterial infection triggers xenophagy to control bacterial replication (in the case of *S. flexneri* infection) and that *S. typhimurium* developed mechanisms to escape from autophagy by reactivating mTORC1 activity.

*Listeria monocytogenes* has also been reported to induce amino acid starvation-induced cellular stress and activation of the GCN2-eIF2α pathway upstream mTOR. Upon de detection of a decrease in the amino acid pool, mTOR activity is reduced leading to autophagy activation in order to normalize this condition. Unlike what is observed during the infection of epithelial cells with *S. flexneri*, in *L. monocytogenes* cells, autophagy is kept repressed, suggesting that *L. monocytogenes* possesses other virulence weapons to block autophagy (93–95) (Figure 7). Finally, AMPK has also been implicated in the enhancement of xenophagy during the infection with *E. coli*. According to this study, *E. coli* infection leads to an increase in intracellular calcium levels, which activates Ca(2+)/calmodulin-dependent protein kinase kinase β (CaMKKβ) to promote AMPK activation. AMPK was undoubtfully implicated in CaMKKβ-mediated xenophagy when macrophages were silenced for AMPK and control of *E. coli* replication was dampened (129).

## Concluding Remarks

Xenophagy has been widely reported to target bacteria for autophagic degradation, with clear impact on intracellular bacterial handling. Even with major advances in our understanding of the mechanisms involved in cargo selection, many questions remain unanswered. For example, why so many different mechanisms to target bacterial pathogens exposed to the cytosol? Still, why different autophagic adaptors and ubiquitin-ligases with apparent redundant functions? Although no evidence in this direction has been reported, we cannot exclude that different types of autophagosomes exist. Thus, p62, NDP52, NBR1, and Optineurin would function as sorters for different autophagosomes. It is possible that the different ubiquitin-ligases work in this direction as well by adding different ubiquitin linkages to the bacterial surface. Regarding bacteria-induced nutritional stress and autophagy induction, it is still to be elucidated whether amino acid starvation is induced upon infection with bacterial pathogens other than *Shigella, Salmonella*, and *Listeria*. Also, why bacteria induce amino acid starvation that leads to autophagy to subsequently inhibit it?

In *in vitro* studies, it is clear that only a fraction of the intracellular bacterial population is targeted to autophagosomes, with modest impact in bacterial replication control following autophagy ablation. This is in sharp contrast to *in vivo* studies, which demonstrate much more pronounced differences in bacterial replication in the absence of autophagy. How would these differences be explained?

Future goals in the field must address these open questions to provide a full understanding of the role of autophagy in bacterial infections.

## Author Contributions

LT, MS, and RM wrote the paper. LT also revised the final document.

## Conflict of Interest Statement

The authors declare that the research was conducted in the absence of any commercial or financial relationships that could be construed as a potential conflict of interest.
